# The challenge of return to work in workers with cancer: employer priorities despite variation in social policies related to work and health

**DOI:** 10.1007/s11764-019-00829-y

**Published:** 2019-11-22

**Authors:** Angelique de Rijk, Ziv Amir, Miri Cohen, Tomislav Furlan, Lode Godderis, Bojana Knezevic, Massimo Miglioretti, Fehmidah Munir, Adela Elena Popa, Maria Sedlakova, Steffen Torp, Dana Yagil, Sietske Tamminga, Angela de Boer

**Affiliations:** 1 Department of Social Medicine, CAPHRI Care and Public Health Research Institute, Maastricht University, Maastricht, The Netherlands; 2 School of Health Sciences, University of Salford, Greater Manchester, UK; 3 School of Social Work, University of Haifa, Haifa, Israel; 4 Adria Medic Occupational Health Clinic, Pazin, Croatia; 5 Department of Public Health and Primary Care, Centre for Environment and Health, KU Leuven, Leuven, Belgium; 6 IDEWE, External Service for Prevention and Protection at Work, Heverlee, Belgium; 7 Department of Quality Improvement in Health Care, University Hospital Centre Zagreb, Zagreb, Croatia; 8 Department of Psychology, University of Milano-Bicocca, Milano, Italy; 9 School of Sport, Exercise and Health Sciences, Loughborough University, Loughborough, Leicestershire UK; 10 Faculty of Social Sciences and Humanities, Lucian Blaga University of Sibiu, Sibiu, Romania; 11 Central European Labour Studies Institute, Bratislava, Slovakia; 12 Department of Health, Social and Welfare Studies, University College of South-Eastern Norway, Notodden, Norway; 13 Department of Human Services, University of Haifa, Haifa, Israel; 14 Coronel Institute of Occupational Health, Amsterdam Public Health research institute, Amsterdam University Medical Centers, University of Amsterdam, Amsterdam, the Netherlands

**Keywords:** Cancer, Cross-country variations, Employer, Return-to-work, Qualitative

## Abstract

**Purpose:**

This study explored employer’s perspectives on (1) their experience of good practice related to workers diagnosed with cancer and their return to work (RTW), and (2) their perceived needs necessary to achieve good practice as reported by employers from nine separate countries.

**Methods:**

Twenty-five semi-structured interviews were held in eight European countries and Israel with two to three employers typically including HR managers or line managers from both profit and non-profit organisations of different sizes and sectors. Interviews were recorded and transcribed verbatim. A grounded theory/thematic analysis approach was completed.

**Results:**

Employers’ experience with RTW assistance for workers with cancer appears to be a dynamic process. Results indicate that good practice includes six phases: (1) reacting to disclosure, (2) collecting information, (3) decision-making related to initial actions, (4) remaining in touch, (5) decision-making on RTW, and (6) follow-up. The exact details of the process are shaped by country, employer type, and worker characteristics; however, there was consistency related to the need for (1) structured procedures, (2) collaboration, (3) communication skills training, (4) information on cancer, and (5) financial resources for realizing RTW support measures.

**Conclusions:**

Notwithstanding variations at country, employer, and worker levels, the employers from all nine countries reported that good practice regarding RTW assistance in workers with a history of cancer consists of the six phases above. Employers indicate that they would benefit from shared collaboration and resources that support good practice for this human resource matter.

**Implications for cancer survivors:**

Further research and development based on the six phases of employer support as a framework for a tool or strategy to support workers with a history of cancer across countries and organisations is warranted.

## Introduction

Each year, 3.4 million new cases of cancer are diagnosed in Europe, of which about 1.6 million are of working age [1]. With improvements in diagnosis, treatment, and survival rates, returning to paid work after cancer diagnosis and treatment has become of increased importance to individuals, employers, and the wider society [2].

Motivations for return to work (RTW) are based not only on financial but also on psychological needs as well, because work often provides meaning to people’s lives [3]. Many studies have shown that work plays an important role for cancer survivors’ identity formation, self-esteem, financial security, and social relationships, and represents capabilities, skills, and health [4]. Although some people affected by cancer are simply able to continue working in a similar manner as prior to diagnosis [5], a significant proportion of them end up unemployed, retire early, or change jobs more often than those without cancer [6–9]. While 63% of 64 studies of cancer survivors returned to work [10], one important reason for not returning to work given by patients is a reported lack of understanding and support from their employers [11, 12]. This was also experienced by other stakeholders including insurance institutes, doctors, and persons in the patient’s private life [13]. Some cancer survivors feel vulnerable and insecure in relation to RTW and are in need of acknowledgement of their concerns by their employers [14]. From an employer perspective, the RTW of knowledgeable and experienced workers enables continuity of skilled manpower and minimizes costs due to lost productivity while reducing payment of disability compensation [15]. While there is research on the perspectives of employers with experience with workers with a history of cancer [12, 16–18], there is a need to determine whether employers experience patterns of useful strategies for RTW across diverse nations.

Evidence suggests that employers have a considerable impact on many aspects of the workers’ wellbeing [19] and that, as expected, employers play a central role in the success of RTW. Employers are in a position to make workplace accommodations, provide support, and facilitate colleagues or co-workers [14, 17, 18, 20, 21]. Cancer survivors are more likely to be employed if they perceived their employer as accommodating and received support from colleagues and employers [22]. Furthermore, given the central role of the employer in all matters related to work in a specific organization, nine out of ten recommendations in the European Declaration call for a guideline on cancer and work involving the employer [23]. Employers however struggle with this role [12, 16] and are in need of support to achieve positive outcomes related to work and cancer survivorship [17, 18].

Therefore, further elaboration of the specific perspectives from various employers in the return to work process of cancer survivors may assist in the process of identifying employer actions that may help improve work outcomes. The aim of the present study is to obtain a better understanding of the employers’ experience with good practices related to employees with a history of cancer and identify specific actions to achieve positive outcomes.

## Methods

The COnsolidated criteria for REporting Qualitative research (COREQ) Checklist was used for reporting of findings [24].

### Sample

Interviews were performed in Belgium, Croatia, Israel, Italy, the Netherlands, Norway, Slovakia, Romania, and the UK, all members of the CANcer and Work Network (CANWON) [25] and representing Eastern, Western, Northern, and Southern European-style welfare state models or a mix of these. Using purposive sampling targeting, three employers per country were approached that together represented the following characteristics: (a) at least two organisations each with small (≤ 50 workers), medium (50–500 workers), and large (> 500 workers) workforce; (b) three different sectors; and (c) both profit and non-profit organizations. Employers were approached via professional networks and publically available contact information. Inclusion criteria for interviewees were (1) employer with experience and close involvement in supporting one or more worker(s) diagnosed with cancer in the last 5 years, (2) at least two employers per country with employees with some experience with a co-worker with cancer who recently returned to work, (3) an organisation that complies with national legislation, and (4) an employer fluent in the country’s language.

### Data collection

Employer representatives (HR managers, line managers) were contacted either by e-mail, telephone, or face-to-face, and informed about the aim of the study and asked to provide written or oral informed consent prior to the interview. The interview guide (see Table 1 for topics) was adapted from Tiedtke et al. [12] with the authors representing the countries. Interviews provided a consistent set of questions across countries.

**Table 1:** Interview guide

Part of the interview	Items and questions
Start	Start with explaining goal, anonymity, confidential treatment of interview data.
Background information	Ask for background information - Inclusion criteria - Age and gender - Function/relation with worker(s) with cancer: line manager, HR manager, other - # workers (small (50), medium (50–500), large sized (larger than 500)) - Type of organisation (sector and being profit or non-profit organisations)
Questions on experiences	1. What does ‘being a good employer’ mean according to you in relation to workers with cancer (with health problems)? (productivity, care, taking care of financial arrangements….) (Next focus on dilemmas, uncertainties, good practices in their experiences) 2. How do you know that a worker has cancer? (privacy, trust, disclosure, communication in organisation) 3. Do you contact the employee, if so, how do you keep contact with the worker? 4. How did it affect your organisation? (replacing, colleagues, clients, money, worker-relation, legal issues, work accommodations, barriers, facilitators) 5. What solutions did you use? (policy available, decision making by who, weighing pros and cons, other actors (trade union, health professionals etc.) involved, barriers (for RTW)) 6. Which solutions would you advice to other employers who have workers with cancer? (refer to actors in answer question 5, successes, failures, invite to be creative, lessons learned) • Interviewer summarizes successful solutions mentioned
Question on needs	7. What kind of resources (info, education, consultancy, financial…) would you want or what do you think other employers would need to achieve successful solutions you mentioned? (tools used, link to what they believe is a good employer)
Ending the interview	Do you have something to add? Would you want to test a tool based on this research? Thank you!

The nine interviewers were researchers or practitioners who were employed in work or health areas and had experienced conducting interviews. The majority of the interviewers and interviewees had no established contact prior to the interview and interviews were preferably completed by one interviewee. Interviews were conducted between May 2016 and March 2017. The interviews lasted 30–60 min, were held in the interviewers’ and interviewee’s native language, and were held face-to-face at the workplace or by telephone/Skype when necessary for logistical reasons. All interviews were audio-recorded and transcribed verbatim if the interviewee agreed. In two cases, a written account of the interview was produced directly after the interview. Interviews were not returned to interviewees for comments. Interviews were translated into English by the interviewer or a co-author from the same country. Data collected and analysed during the study are available from the corresponding author upon request.

### Sample characteristics

In total, 59 employers were approached (range 3–30 per country) and 25 interviews were conducted including 27 employer representatives (response rate 42%). Average age of the interviewees was 48 years, 10/27 were female, 13 were directors or line managers, and 14 HR or health and safety professionals. The sizes of the organisations varied between small (*n* = 5 employers), medium (*n* = 9 employers), and large (*n* = 11 employers). Diverse sectors with 6 companies from the industry sector were represented. Fourteen organisations were for-profit organisations, while the others were non-profit and mostly public organisations (see Table 2).

**Table 2:** Characteristics of interviewees

	Country/int#	Age	Gender	Function	Company size	Non-profit/profit	Sector
1	Belgium 1	56	m	HR director	Large	Non-profit	Health and social work
2	Belgium 2	49	m	Regional director	Large	Non-profit	Health and social work
3	Belgium 3	47	f	Line manager	Medium	Profit	Manufacturing
4	Croatia 1	56	m	Trade union director	Small	Non-profit	Education
5	Croatia 2	35	m	OHS expert	Medium	Profit	Wholesale and retail trade
6	Croatia 3	52	f	OHS expert	Large	Non-profit	Public administration and defence
7	Italy 1	53	m	HR director	Medium	Profit	Manufacturing
8	Italy 2	60	m	CEO	Small	Profit	Science
9	Italy 3	50; 58	m; m	HR and OHS directors	Large	Profit	Manufacturing
10	Israel 1	45	f	Department manager	Large	Non-profit	Education
11	Israel 2	53	m	Department manager	Large	Profit	Manufacturing
12	Netherlands 1	44	f	HR manager	Small	Profit	Education
13	Netherlands 2	48	m	Line manager	Medium	Profit	Transportation and storage
14	Netherlands 3	54	f	Line manager	Large	Non-profit	Health and social work
15	Norway 1	44	m	Line manager	Large	Profit	Construction
16	Norway 2	55	m	Administrative director	Medium	Profit	Maritime industry
17	Norway 3	55	f	Principal	Small	Non-profit	Education
18	Romania 1	53	m	HR manager	Small	Non-profit	Public administration
19	Romania 2	45	f	HR manager	Medium	Non-profit	Health and social work
20	Romania 3	38	m	HR consultant	Large	Profit	Manufacturing
21	Slovakia 1	35-40	f	HR director	Medium	Profit	Manufacturing
22	Slovakia 2	28	f	HR manager	Medium	Profit	Information/communication
23	Slovakia 3	40-50	f; m	HR and Health managers	Large	Profit	Manufacturing
24	UK 1	N/A	m	Director	Large	Non-profit	Education
25	UK 2	N/A	m	Manager	Medium	Non-profit	Health and social work

### Data analysis

A mix of grounded theory [26] and thematic analysis [27] was adopted, emphasizing collaborative analysis from different country perspectives; all themes were derived from the data and none were identified in advance. All interviews (which had been translated to English, see above) were coded, and a preliminary overall schematic representation of the interviews was provided [28] by the first author. Next, all authors read the interviews completed in their own country in addition to at least two from another country, produced narrative reviews in English of these interviews, and went through a process of rough coding. A meeting with most authors in Milan, October 2016, was used to collaboratively define preliminary themes for a more refined coding.

Then more specific coding on the basis of themes (top down) and data (bottom up) was performed and a first version of results was produced. The findings were discussed at a second meeting with authors in Loughborough, January 2017, and a refined overall schematic representation of the interviews was produced. A second version of the results was produced based on this meeting.

Finally, in a third meeting with authors in Bratislava, March 2017, results were discussed and the final overall schematic presentation of the interviews was established. A final version of the results was agreed upon via e-mail.

### Ethical issues and confidential treatment of data

All procedures performed involving human participants were in accordance with the ethical standards of the institutional and/or national research committee and the 1964 Helsinki Declaration and its later amendments or comparable ethical standards. Ethical approval was assured and in line with national standards in each country.

All interviewers informed the participating employers and asked for their written or oral informed consent and all transcripts were anonymous. The researchers who read, during the process of data analysis, the anonymous interviews from another country, all signed a statement that they will treat the transcripts confidentially. Each interviewer who collected the data was held responsible for treating these data according to national standards.

## Results

**Fig. 1:**
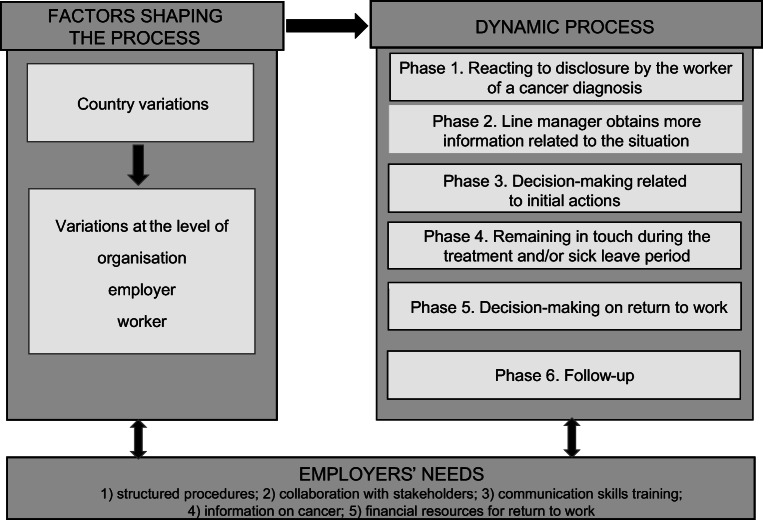
The dynamic process of returning to work after cancer from the employers’ perspectives

Overall, employers viewed the experience of assisting a worker with a history of cancer in relation to RTW as a dynamic process. Six support phases were identified as good practice for addressing RTW of a worker with a history of cancer. The support differed in level of comprehensiveness by country, organisation, employer, and worker. However, the dynamic process implicated by the expressions of employers across these nations is summarized in Fig. 1.

### Dynamic process

Rather than a procedure that can be simply managed, employers referred to supporting workers as a process depending on the specific context.

*“But what I always find important it that it is a process between line manager and worker (…).”*

As indicated in Fig. 1, this process typically involves six different phases: (1) reacting to disclosure by the worker, (2) line manager obtains more information related to the situation, (3) decision-making related to initial actions, (4) remaining in touch during the treatment and/or the sick leave period, (5) decision-making on RTW, and (6) follow-up. This dynamic process does not always follow a stepwise method where phases are not always addressed in the order described and can be brief or comprehensive. Employers tend to tailor the process to the situation and person. The needs of a specific worker are considered and flexibility is a major priority.

*“(…) A good employer should pay particular attention to a serious illnesses like cancer, in order to manage working time and required tasks in relation to the worker. Here’s what I think: (…) the organisation (…) absolutely has to consider every local situation and people working in the enterprise.”*

#### Phase 1: Reacting to disclosure by the worker of a cancer diagnosis

Involvement of the employer is only possible if the cancer diagnosis is disclosed, which is not mandatory according to privacy legislation across all nations. Most workers do explain their situation to their employer. Alternatively, the HR department, the occupational physician, or the family might discuss the situation with the line manager, emphasizing confidentiality of the information.

*“(…) the Human Resources department will normally contact the head of department (…) to let them know.(…) within that, there’s advice about not sharing this information (…)”*

The employers consider a worker’s cancer diagnosis to have a special status compared to other health conditions, for three reasons. Firstly, the diagnosis is not questioned. Secondly, “*With cancer, one thinks immediately of the risk of death, and that is what makes it special (…)”* and thirdly, cancer is regarded as psychologically demanding for the worker. As a consequence, employers often mention that the diagnosis of cancer facilitates workplace support.

*“I believe that cancer is creating something extra among all the persons around this person (…) compared to (…) musculoskeletal disorders. It [cancer] creates (…) a greater will to contribute (…) and patience in relation to the unpredictability.”*

#### Phase 2: Line manager obtains more information related to the situation

After the cancer diagnosis is disclosed, the employer—mostly the line manager—begins to obtain relevant information needed for early decision-making, either in the same meeting during which the disclosure took place or in additional meetings. In some countries, an occupational physician meets the worker and delivers the information needed. The aim of this phase is to gain knowledge on the functional consequences of medical situation, the treatment and the psychosocial situation. Also, contraindications for work at the specific workplace is checked (e.g. amount and type of work demands), and—if appropriate—determination of whether the commuting time remains feasible. This phase requires sensitive communication skills and the willingness to invest time in such discussion.

*“It is important to keep a bond with the worker with cancer and to be there for that worker. Investing time for this is essential. You should cope with it consciously*.”

Often, employers wait with decision-making until the medical assessment has been completed and the course of treatment becomes clear. If access to health care becomes a problem, employers might help the worker to obtain medical care sooner.

#### Phase 3: Decision-making related to initial actions

After a first impression is made of a worker’s situation, employers decide whether or not the worker will continue to work during (waiting for) treatment and if so, under what conditions. Four types of this initial decision-making process were reported.

Firstly, the worker might continue working in the same job under the same conditions. This might involve redistribution of specific tasks within the team, e.g. strenuous work tasks are replaced by more simple tasks when the worker’s energy level is low because of chemotherapy. Furthermore, some flexibility from the line manager regarding working times can be required, but this might not be possible when the affected worker is the manager himself.

*“(…) he had to work, because you cannot just not show up if you are the boss. (…) He worked the entire time, while I was looking for information or doctors. (…) It was all physically demanding for him too.”*

Secondly, the worker might continue working in adapted work. For example, shift work may be suspended. Thirdly, the worker might continue working in a different job with a different contract. Continuing to work can be regarded as beneficial for the worker, yet it is more of an exception than a rule.

Fourthly, the worker may be given sick leave by the occupational or treating physician (depending on the country’s jurisdiction). Then, an important employer issue is the decision to replace the worker or not. Some employers suggest that not replacing the worker represents good practice. According to them, replacement might be perceived by the worker as a sign that the job is no longer available for them and that the employer might possibly not want him or her back because of impending mortality. However, most employers indicate that they prefer to avoid such expectations.

*“I would like to add, he [the worker] simply must have the feeling that he has a place to return to, [and not the feeling] that he is not valued within the company and that his position will be occupied by someone else.”*

This phase of initial decision-making causes employers to struggle with diverse dilemmas regarding informing and guiding the worker’s colleagues. Firstly, they feel responsible for safeguarding the privacy of the worker. Secondly, there is the emotional reaction of the team and this also requires the employer to take time for communication.

*“There is an important impact on the person and on the team. One has to be on ‘stand by’ for one’s workers.”*

Thirdly, lack of replacement for the affected worker might be a burden to co-workers. In such cases, employers report using their management/leadership skills to create goodwill in the team, at least for some time.

#### Phase 4: Remaining in touch during the treatment and/or sick leave period

All employers try to keep in touch with their affected workers in different ways and with different frequencies. There might be meetings at home or in the organisation, or telephone calls. The line manager, HR professionals, or occupational physicians might be involved. The goals of this problem solving includes staying in touch with the worker in order to ensure that a worker’s needs are clear and to collect necessary information on the course of the illness and the treatment via the worker.

In some countries, a formal plan that for example specifies proposed work adaptations, counselling, meetings with the line manager and occupational physician, and the organization of phased RTW is required. This plan is written up by the employer at an early stage and adjusted as needed, and requires regular meetings.

*“HR is really strict on providing a plan. There are routines on that etc. They send out questions that are supposed to be responded to and there must be a written follow-up plan every fourth week. For example if it is due on 26*^*th*^*, they follow up soon after.”*

#### Phase 5: Decision-making on RTW

When the effects of treatment on the worker’s work ability become clear, a more lasting decision for the near future needs to be made. At this stage, it is not only the employer (line manager and or HR professional) who decides but depending on which stakeholders are available in a country; occupational physicians, a psychologist in the organisation or community, social workers, social insurance, and trade unions can also be involved. Essentially, five possibilities were described by employers:

1. The worker’s workability is too low and the worker applies for financial compensation, if available.
2. The worker is on a temporary contract that expires during treatment. The worker is either out of a job and the involvement of the employer ends or the employer evaluates the situation and tries to identify a job for the worker.
3. The worker himself or herself decides not to continue working. The employer may find this understandable given the personal situation of a worker.
4. The worker returns to work part-time, and still receives some social welfare. The number of work hours may increase over time (depending on whether legislation allows gradual RTW).
5. The worker returns to work full-time, possibly with adjustments including if feasible the option of working from home, to reduce demands. If a worker returns to work, the employer often feels responsible to remove work-related risks that might negatively affect the worker’s health. Often, returning to work full-time includes a process of gradually increasing working hours until the worker is back full-time (if legislation allows gradual return to work):

*“(…) At a certain time, the situation was hardened. Because, yes, we said, well, you have been ill for a year, we are going to ‘track 2’, that means, we have to re-integrate you, thus you have to build up again. That means that you will return to work slowly, from 20 hours to 22, 23, 24.”*

#### Phase 6: Follow-up

Employers emphasize that they cannot control the outcome of the cancer treatment. Some employers report that workers who returned to work after cancer are still considerably vulnerable, which the employer interprets as coming from the psychological stress of cancer and its treatment.

*“It has been three months since she got back (…). The stress was enormous, she was stressed but never cried, she functioned but was very stressed, she wanted to know the results (…).”*

Some employers explain that despite their efforts, RTW appeared not possible because of what they referred to as the most difficult outcome: death of a worker. These employers explain that this was a learning experience for them and they warn that employers have to be prepared for this adverse outcome, particularly because of the negative impact it has on the team.

*“And the big difference is when it does not go well. When death comes. Because then you are stuck in some kind of a vacuum. And how do you cope with that? (…) (…) there is a wider dimension (…), and that’s the interpersonal relationships.”*

For this outcome, there are no protocols, but some employers refer to the important need of support for the line manager and the whole team, and financial support for the remaining family.

### Factors shaping the RTW process

According to the employers interviewed, how RTW processes are actually detailed for the affected worker is still a function of policy related to country, organisation, employer, and worker.

#### Country variations

Even though income protection for the work disabled differs across countries, all employers indicated they try to safeguard income either by work or welfare. However, large country differences in the degree of employer involvement in the different RTW phases exist. Policies vary from scarce sickness absence policies in for example Romania and Slovakia, to detailed sickness absence policies such as in Norway and the Netherlands, which require employers to take a large role.

In countries with scarce national policy on sickness absence, employers choose either taking the primary responsibility or expecting that worker to take the main responsibility. Employers taking the main responsibility might focus on the detailed planning of RTW, as in the following quote.

*“(…) we are (...) more able to manage it, maybe because we face very different situations since the health conditions and domestic situations are different for each case. Since we are in charge, we have to exactly plan how the situation will develop (...).”*

Such employers might also focus on organizing (medical) support.

*“(...) maybe in the sense that if he needs help we (…) can help with some contacts, help in speeding up the examination at the doctors, or maybe so that he receives the best health care that can be provided.”*

Other employers in countries with scarce policy emphasize being compassionate, i.e. understanding, empathic, or giving space to the worker diagnosed with cancer, and thereby leaving the responsibility to the worker.

*“If they are close to their workers they will find the best solutions. So it depends on the employer…. If he wants to help he will eventually find the legal and organisational ways to help the worker. What matters is the human side.”*

In contrast, in countries with more extensive national policy on sickness absence, mutual investment by both the employer and the worker is expected.

*“Thus what we always do is look at the full package of tasks, but I let the person think about it. The person has to indicate to me: this I will not be able to do, that I will be able to do. And we try to facilitate that (…) you have to get people in the mode that they think about this themselves.”*

Employers in these countries describe the process more precise and in terms of their duties for realizing work accommodations and planning gradual RTW.

#### Variations at the level of the organisation

Within the margins and requirements of national legislation, organisations also vary in the level of comprehensiveness of their sickness absence policies and what these policies require employers to do. These policies vary from a strict policy prescribing employers what to do in different phases to employers deciding themselves on ad hoc procedures:

“*There is a sickness absence protocol. The employer has to contact the worker every 14 days.”*

*“(…) we don’t have a procedure about calling the worker at home and contacting them on certain dates, and so on (…). (…) It stays at a personal level, how each of us knows to manage it; I say it’s about feeling (…)”*

Also, the size of the organisation matters. Employers of smaller companies express a of lack organisational policies but pictured their organisation as families with strong ties between the workers. They have great concerns over the risk of losing their reputation and thus confidence of the consumer, who often live in their direct environment. Job turnover is rare. Employers of larger organisations express to have routine systems in place to manage workers with cancer, and financial buffers and options to dilute the burden for colleagues. They have well-developed organisational sickness absence policies and most have a socio-medical team. However, the flexibility of larger organisations is sometimes compromised by hierarchical decision-making. The RTW process is thus more unpredictable and flexible in smaller organizations, and might be more extensively controlled in larger organisations.

Irrespective size or sector, employers refer to different social cultures in organisations. Some refer to an empathic culture, in which during each phase, the worker with cancer is monitored and receives a tailored approach:

*“It was less about, look, here’s a policy and do it by numbers. It was more about actually how are we going to get the best out of this person now and support them in their recovery……let them feel that, without becoming sentimental, let them say, look, we care about your wellbeing,(...)*.”

Others refer to strictness and maintaining the competitiveness of the organisation:

*“Brutality is in things and in situations, especially when the system of costs becomes very strong, as in multinational corporations: if somebody has a cancer, a worker in HR offices calls him and gives him a redundancy pay and fires him.”*

Finally, some employers explained that RTW is more complicated if during the sickness absence important organisational changes take place, such as a merger with another organisation.

#### Variations at the level of the individual employer

The RTW processes also vary because the employers are different people, with different skills, experiences, and characteristics. Some employers emphasize that their acquired leadership qualities and communication skills had made them fit for the job of guiding a worker with cancer, and they invest more effort in each phase.

*“It was more about my leadership experience and treating someone just sensitively. So I had the ability to say to M., let’s start with you coming back three days a week and then we’ll work from there, and if you want to come in a little later (…)”*

Other employers have experienced cancer in their personal lives or with other workers, and feel that because of these experiences, they feel more secure in the decision-making.

*“I knew as I had my mother in that situation.”*

Finally, the employer’s personality, *“me as a human being”*, influences the extent to which the employer feels emotionally affected during the RTW process, varying from protecting themselves against becoming too emotional, phrased as *“not go down the basement”* to being *“affected so much”*.

#### Variations at the level of the worker

Finally, and within the context of national and organizational policy, most employers take characteristics of the worker into account when deciding about work adjustments and other types of support. Workers differ regarding their disease characteristics, personality, and work motivation; their position in the group; and in their relationship with the employer before the cancer diagnosis. Workers also differ regarding their emotional reaction and family support. Generally, workers who are more positively appreciated by their employer can rely on more employer support.

*“(…) not married, (…) and (…) her life revolved around university and also the team here, (…). So, she was much more keen to try and come into work. So, that was a slightly different approach (…).“*

Finally, workers in lower job levels can be replaced more easily when compared with workers with heavy responsibilities. The results presented above identified employers’ experiences of good practice when assisting a worker with cancer as a phased process, shaped by factors at country, organization, and employer and worker level.

### Employer needs

Employers report five types of needs across the different countries in order to better achieve good practice in relation to a worker with cancer.

Firstly, employers express the need for structured procedures.

*“And I am in favour of procedures and rules and guidelines.”*

As the previous quote illustrates, it is about rules and legislation. Some employers refer to legislation specific for cancer patients to fulfil this need.

“*Perhaps legislation could help us more. (…) For example, the legislation should mention: if they return to work what you have to do, what one should not do, etc. (…).”*

Another option discussed is to have structured procedures by trade unions in collective bargaining. Furthermore, it is about organisational policies, which importance is expressed by employers from countries without and with extensive sickness absence legislation and policies:

*“I think it would be very useful to establish return to work procedures after long sick leave.”*

*“(…) we have, and most of us [organisations] have systems to handle it [sickness absence]”. (…) For handling people on sick leave, you must have systems.”*

Finally, employers express the need for professional guidelines and protocols, based on the literature and knowledge from practice.

*“The employer needs clear instructions on what the workers can and cannot do, so that they can adjust their workplaces or give them another job.“*

*“I would appreciate you forming a working group who collects experiences and knowledge to formulate a guideline.”*

Secondly, many employers emphasize collaboration with other stakeholders, among which the role of the occupational physician or occupational health specialist is highly appreciated.

*“(…) you have to tune things with the social-medical team, and you need to understand well the responsibility of the occupational physician and of the line manager. We have our P&O department involved in the line management (…) and the HR manager should know and take his responsibility (…) And I think as being from the P&O department that he should not check but monitor [the process].“*

*“The role of the occupational health specialist is very important. The occupational health specialist could come into the company and visit each workplace. The occupational health specialist makes work ability assessment and draws up an expert opinion about job tasks in a certain company about what workers can perform and what cannot be performed anymore.”*

Thirdly, most employers refer to communication skills training.

*“A more focused training would be good, or to communicate with such workers from the perspective of the employer.”*

Fourthly, they also like to get information on cancer and its treatment side effects, the capabilities of the worker who returns and possible work adaptations. With such scenarios of a worker with cancer, then they are better able to deal with the diverse dilemmas and the emotional burden they experience as an employer. This can be done via consultation with experts or an easy-to-access information package according to employers from diverse countries:

*“Maybe if there was some ‘package’ prepared, which would summarize all the alternatives in cases of workers with cancer, to indicate that one doesn’t need to experience all of it alone. (…) something simple. Which can be downloaded from the Internet and then distributed to line managers and colleagues, to see what the different possibilities are for how it develops.”*

Fifthly, employers refer to the need for financial resources for them to assist workers with cancer.

*“I think NGOs could help financially or in another way. We do not have a union but in other sectors maybe unions could engage and help in these issues.”*

In some countries, the social insurance supports financially.

*“We also realized the person could work up to 20% without payment from the company. Social insurance paid this just to try to get him going and that stimulates.”*

These needs fit with the finding that the process of guiding a worker with cancer is perceived as a dynamic process consisting of several phases, requiring planning, collaboration, sophisticated communication skills, diverse knowledge, and financial resources.

## Discussion

The aim of this study was to gather different employer perspectives on workers with cancer including employers’ experiences of good practice and their perceived needs to support good RTW outcomes in these workers. A novel aspect of this study is that employers were from nine different countries with different welfare systems.

Despite variations across countries, six phases were identified by employers with corresponding needs for employer support. The first phase encompasses the employer’s initial reaction to the diagnosis. While disclosure of cancer to an employer is not demanded by law in participating countries, employers felt it was essential to be informed so that decisions such as temporarily re-allocating the worker’s job tasks could be made.

Following disclosure, employers gather relevant information (phase 2) to help them to decide about initial action (phase 3). While employers used their general managerial skills to gather relevant information from a worker diagnosed with cancer, they expressed the need for more sophisticated communication skills. The emphasis on advanced communication skills is in line with previous research in employers managing workers diagnosed with cancer [16, 17]. During treatment and/or sickness absence, the employer remains in touch with the worker (phase 4), again requiring good communication skills. When a worker’s RTW becomes a possibility, decisions are made by employers related to RTW and adaptations, and further planning is needed (phase 5). Finally, employers address follow-up of RTW or, in the worst case, of dealing with advanced, non-curative disease (phase 6).

Employers’ views on good practice are in line with the scientific literature on how to organize RTW for cancer patients, including involving problem assessment (including both the worker and the workplace), planning, performing planned activities, and evaluation [29]. However, the current study shows employers put greater emphasis on managing initial actions and sick leave than on actual RTW. This is similar to the finding among Belgian breast cancer survivors, which shows that an intensive mental preparation by workers preceding actual RTW is important [13]. A Dutch study on employers managing sick leave and return to work among workers with cancer reported five phases that reflected national legislation. However, the current study did not explicitly distinguish a phase for early information gathering [16].

Collaboration with stakeholders was also expressed as a need particularly when it comes to designing RTW. Occupational health physicians, for example, are trained to translate medical problems into functional issues and fitness for work. Generally, RTW is regarded a multifaceted process involving many stakeholders [13–17]. Overall, employers in the current study expressed a need for structured procedures consistent with earlier recommendations [18].

The consensus on the phases and needs between the interviewed employers across countries and organisations is clearly striking, particularly given differences between countries on whether employer involvement in RTW is required by legislation as indicated in ‘variations at level of organisation’. The intensity by which the six phases are addressed was more intense among employers from those countries that have well developed national RTW guidance or legislation. However, the current study did not identify ethical dilemmas faced by employers as found in previous studies [12, 16] such as feeling caught between their sincere concern for the employee and the professional realism of interests of the company. This might be explained by the focus on good practice in the current study.

## Strengths and limitations

This is the first study to explore the experiences and needs of employers regarding workers with cancer across countries representing diverse welfare models [30]. We selected a wide variety of employers from different backgrounds and different sizes and types of companies. All interviewers used the same interview guide developed collaboratively by the authors, which increases validity of the data. In addition, during the analysis, three day-long face-to-face meetings took place with the interviewers and data coders (if different) to increase validity of the analysis [26, 28].

This study has several limitations. Some employers were selected via professional contacts of the interviewers, and about half of the employers approached did not participate. Therefore, selection bias towards a general positive view on the workability of cancer survivors, both via selection of employers and via selective report of employers in order to present a positive image, cannot be ruled out. This is a general difficulty in research on RTW among employers [12, 16, 18, 20]. Indeed, results from survivors’ perceptions show that they are not always positive on the role of the employer and they can perceive barriers related to support, communication, work environment, discrimination, and perception of work ability [17].

The strict selection criteria leading to a balanced composition of employer types (with also representation of small organisations) might have counterbalanced this limitation at least partly. Moreover, we had a response rate of 25/59 of the approached employers which can have caused a positive selection bias as well. In addition, a total of 25 interviews might not be many given that nine countries were involved, but it is not a low number for qualitative research. Further, although additional information could have been provided by additional employers, the consistent findings across countries might be regarded as a sign of data saturation. Cross-country variations might have impacted the data from the interviewer’s part. Translation to English might have led to important linguistic nuances being lost in translation. This last disadvantage has been counterbalanced by opportunities for reviewing results by all interviewers. To reduce demands for employers, they were not asked for any further comments after the interview questions. To conclude, the findings might not represent all good practice experienced in each of the nine countries, but certainly represent an important part of the employer experiences.

It may not be possible to generalize the findings to countries not included and countries outside Europe or Israel. Reliance on the presence of benefits for some level of income replacement for a worker on sick leave might be the explanation for the high degree of consensus among employers.

## Recommendations for further research

Quantitative studies are also needed to substantiate and further determine the generalization of these findings. It is important to develop, implement, and study the effectiveness of interventions aimed at supporting employers in order to enhance sustainable work participation of workers with a history of cancer. Effective interventions for this patient group remain scarce [31]. Finally, more detailed cross-country studies are needed as other studies do show cross-country variations in labour participation of vulnerable groups [33–37].

## Recommendations for practice

In order to improve the process of RTW in cancer survivors, employers need support as well [20]. The framework of six phases found in this study provides policymakers and employers with a tool to organize new national legislation, company policy and individual guidance.

Legislation on work adaptations and gradual RTW can aid employers in supporting RTW of cancer survivors. Across jurisdictions, there is an increasing focus on supporting individual participation in the labour force [32]. Finally, our findings indicate the need for training in communication and decision-making skills of employers related to this problem and perhaps chronic illness and work in general. Caring for workers with cancer should be continued beyond the hospital walls and beyond recovery of the malignancy. Employers are key to realize this.
